# Headache in epilepsy: A prospective observational study

**DOI:** 10.1002/epi4.12363

**Published:** 2019-10-21

**Authors:** Mark A. Whealy, Anna Myburgh, Tanya J. Bredesen, Jeffrey W. Britton

**Affiliations:** ^1^ Department of Neurology Mayo Clinic Rochester Rochester Minnesota; ^2^ Division of Headache Medicine Mayo Clinic Rochester Rochester Minnesota; ^3^ Division of Epilepsy Mayo Clinic Rochester Rochester Minnesota

**Keywords:** epilepsy, headache, migraine, seizure

## Abstract

**Objective:**

To assess the frequency and characteristics of interictal and postictal headaches (using International Classification of Headache Disorders, 3rd edition criteria) in a population of patients with epilepsy admitted to the Mayo Clinic Rochester epilepsy monitoring unit and assess their localizing value.

**Methods:**

This was a cross‐sectional study. Participants were voluntarily recruited upon admission to the epilepsy monitoring unit. Two separate questionnaires were then administered. The first was to assess the presence and character of headaches experienced in the past 12 months. The second was to assess characteristics of postictal headaches experienced during their admission including localization.

**Results:**

One‐hundred and twenty subjects (77%) met inclusion criteria and completed the initial questionnaire. Mean age was 38.1 years (range 18‐82), and 67 (55.8%) were female. Interictal headaches were reported in 97 of 120 (81%) subjects, and these met ICHD3 criteria for migraine in 48 (50%). Postictal headaches were reported by 75 of 120 (63%) subjects on the initial admission questionnaire, representing migraine in 38 (51%). Thirty‐nine (32%) subjects completed the secondary questionnaire related to postictal headaches experienced during admission, of which nine (23%) met criteria for migraine. There was no seizure lateralizing or localizing value noted based on postictal headache localization.

**Significance:**

Migraine was frequent in this cohort and appears to be the dominant interictal and postictal headache type in patients with epilepsy. In this study, the first to assess incident postictal headache in the setting of an ictal EEG, headache localization was of no seizure localizing value. Few patients were being actively treated; suggesting headache management is often overlooked in the epilepsy population.

**Key Points**

- Interictal and postictal headaches are common in patients with epilepsy
- Migraine is the dominant phenotype of interictal and postictal headaches
- In the first study to assess incident postictal headache in the setting of an ictal EEG, there was no localizing value to postictal headaches
- Similar to the general population, headaches were more common in female subjects

## 1 INTRODUCTION

Headache and epilepsy are both common worldwide. The association between headache and epilepsy has been recognized for at least a century.^1^ Headache is an under‐recognized and under‐treated symptom in persons with epilepsy (PWE).^2^, ^3^ Worldwide, an active headache disorder is reported in about 46% of the population. Overall, migraine represents about 11% of headache. Chronic daily headache is seen in 3%, and tension‐type headache is seen in 42%.^4^

The epidemiology of headache in epilepsy has been extensively studied. In reviewing the literature, 7%‐57% of patients with epilepsy report active interictal headaches. Migraine is seen in 2%‐26.3% of this population, while tension‐type headache is seen in 9%‐19.1%.^5^, ^6^, ^7^, ^8^, ^9^, ^10^, ^11^, ^12^, ^13^, ^14^ Despite this literature evidence, the comorbidity headaches in epilepsy is controversial. This makes continued effort to increase awareness in the healthcare community very important.

Postictal headache is reported frequently in PWE. These headaches generally have features of migraine (21%‐53% of postictal headaches).^3^, ^5^, ^6^, ^8^, ^10^, ^12^, ^14^, ^15^, ^16^, ^17^, ^18^, ^19^, ^20^ The limited literature on the lateralizing value of postictal headache localization is mixed, some showing an ipsilateral lateralizing value,^12^, ^17^ while other studies showing no lateralizing value.^11^, ^18^, ^19^ None of these previous studies used ictal EEG.

In this study, we aimed to measure the frequency of headache as a co‐morbid disorder in patients admitted to our epilepsy monitoring unit (EMU) and to clarify the interictal and postictal headache classification experienced in this setting using International Classification of Headache Disorders, 3rd edition (ICHD 3) criteria. An additional aim was to evaluate the localizing and lateralizing value of postictal headaches captured during the epilepsy monitoring unit admission as correlated with EEG and clinical localization.

## 2 METHODS

### 2.1 Subjects and study design

Subjects were recruited from the Mayo Clinic EMU over a period of sixteen months (February 2016 through June 2017). Each subject was given two questionnaires. The first inquired about interictal and postictal headaches experienced in the previous 12 months. The second was administered to all subjects to characterize the prevalence of postictal headaches experienced during the EMU admission, including laterality of the headache with respect to the ictal onset. This second questionnaire was administered by staff in the EMU at the time the headache was occurring. The inclusion criteria were as follows: patients admitted to the adult EMU for evaluation of known or suspected epilepsy who were willing to complete the questionnaire. Exclusion criteria were as follows: lack of an epilepsy diagnosis by EMU evaluation. ICHD three criteria were used to define headache diagnoses (Migraine, probable migraine, tension‐type headache, probable tension‐type headache, postictal headache, etc), and the criteria were used to construct the questionnaires.^21^ Refractory epilepsy was defined as continued seizures despite 2 or more adequate trials of antiseizure medication. Those not meeting this definition were considered nonrefractory.

### 2.2 Statistical methods

Descriptive statistics (number, percent, mean, range) were used to characterize the sample. Pearson's chi‐squared test was used for statistical analysis of categorical variables.

### 2.3 Standard protocol approvals, registrations, and patient consents

The protocol was approved by the Mayo Clinic Institutional Review Board. Written informed consent was obtained from all participants (or guardians of participants) in the study.

## 3 RESULTS

### 3.1 First questionnaire (headache history in last 12 months, Figure S1)

**Figure 1:**
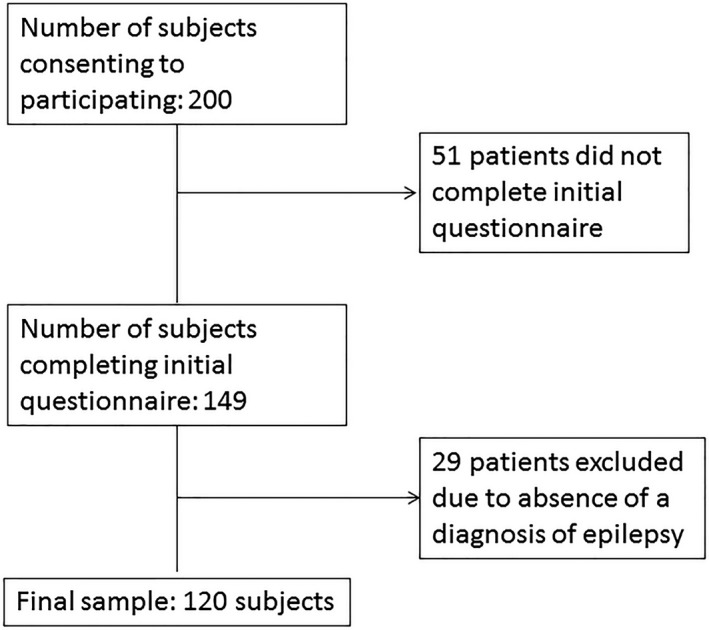
Patient selection flowchart

One‐hundred and twenty patients were included after applying exclusion criteria (Figure 1). Mean age was 38.1 years (range 18‐82), and 67 (55.8%) were female. Interictal headaches were reported in 97 (81%). ICHD 3 criteria for definite migraine were met in 22 (18%) and probable in 26 (22%). Another 14 (12%) and 13 (11%) met ICHD 3 criteria for definite and probable tension‐type headache. Three patients had secondary headaches (intracranial neoplasm, arteriovenous malformation with hemorrhage, and limbic encephalitis). Headache type was not classifiable in 22 (18%). Therefore, definite or probable migraine was present in 48/97 (50%) patients reporting headaches. Females were more likely to report interictal headache (Table 1); however, there was no difference between males and females with regard to reporting of interictal migraine. There was no difference in interictal headaches based on antiseizure medication refractoriness (Table 2).

**Table 1:** Comparing interictal and postictal headaches in males and females

	Male, n (%)	Female, n (%)	*P*‐value
Total	53 (44)	67 (56)	
Interictal headaches, Total	36 (68)	63 (91)	.001
Definite and probable migraine	16 (44)	32 (51)	.45
Chronic daily headache (> 15 headache days per month)	8 (15)	10 (15)	.98
Postictal headaches, Total	30 (57)	45 (67)	.24
Definite and probable migraine	13 (43)	25 (56)	.3

**Table 2:** Comparing Interictal and postictal HA in refractory and nonrefractory epilepsy

	Refractory Epilepsy, n (%)	Nonrefractory, n (%)	*P*‐value
Total	79 (66)	41 (34)	
Interictal headache	62 (78)	35 (85)	.36
Definite and probable migraine	31 (50)	18 (51)	.89
Chronic daily headache (≥15 headache days per month)	12 (15)	9 (22)	.36
Postictal headache	51 (65)	24 (59)	.52
Definite and probable migraine	24 (47)	14 (58)	.36

In the definite/probable migraine subgroup (n = 48), it was noted that 8 (16.7%) were on triptan abortive therapy at the time of enrollment. Another 13 (27%) were on some form of a migraine prophylactic medication when assessing their medications at enrollment. Only 6 (12.5%) reported that they were on medication for prevention of their headaches. The prophylactic migraine medications used in this population included gabapentin (n = 3), topiramate (n = 3), divalproex sodium (n = 3), verapamil (n = 1), metoprolol (n = 1), atenolol (n = 1), riboflavin (n = 1), venlafaxine (n = 1), and cyproheptadine (n = 1). Two subjects were on two migraine prophylactic medications.

Seventy‐five (63%) subjects reported having at least one postictal headache in the previous 12 months. Per ICHD 3 criteria, postictal headaches were defined as headaches occurring within 3 hours of the termination of the seizure. Postictal headaches were classified as definite migraine in 15 (13%) and probable in 23 (19%). Another 10 (8%) and 3 (3%) subjects were classified as definite and probable tension‐type headache, respectively. In the remaining 24 (20%) subjects, the headaches could not be classified based on available information. Therefore, definite or probable migraine constituted 51% of reported postictal headaches. There was no difference between the reporting of postictal headaches or migraine based on gender (Table 1) or antiseizure medication refractoriness (Table 2).

### 3.2 Second questionnaire (incident postictal headache, Figure S2)

A total of 39 subjects experienced 43 postictal headaches during their admission. Nine of 43 (21%) headaches were classified as migraine. The headache was ipsilateral to ictal onset in 8 of 40 (20%) seizures, contralateral in 4 (10%), and bilateral in 23 (58%). The laterality was unknown in 5 (13%) either due to unclear seizure localization or lack of reported headache laterality. In temporal lobe seizures, the headache was ipsilateral in 7 of 22 (32%) seizures, contralateral in 3 (14%), bilateral in 10 (45%), and unknown in 2 (9%). It was noted that in the 39 subjects completing the second portion of the study, only three females and one male were on preventative medications for headaches, and only one male and one female were on headache abortive treatment.

## 4 DISCUSSION

This study supports previous observations as to the high prevalence of headaches in PWE. It still remains controversial if the correlation of epilepsy and migraine is due to shared pathophysiology or merely a reflection of the high general prevalence of migraine. However, the proportion of patients with migraine in this cohort exceeded that of the general population, providing support for the hypothesis of shared pathophysiology. Shared pathophysiology is likely explained by cortical hyperexcitability. Cortical excitability was measured using transcranial magnetic stimulation in patients with epilepsy, migraine only, and controls. In this study, increased cortical excitability was seen in both PWE and in those with migraine only as compared to controls.^22^ Epilepsy is part of the phenotype seen in some of the mutations involved in the familial hemiplegic migraines, FHM1 and FHM2. The mutations involved in these lead to neuronal hyperexcitability.^23^ The different expressions of hyperexcitability in migraine and epilepsy (hypersynchronous cortical activity in epilepsy and cortical spreading depression in migraine) may be related to other factors which have not yet been identified.^24^

We reviewed the literature on the prevalence of headache and migraine in epilepsy populations. The prevalence of interictal headache (81%) and migraine (41%, when combining definite and probable) in PWE in this study were higher than what has been reported in the literature. In the author's review of the literature (Table 3), interictal headache has been reported in 7%‐57% of patients.^5^, ^6^, ^7^, ^8^, ^9^, ^10^, ^11^, ^12^, ^13^, ^20^ One study reported active headache in 92% of patient with epilepsy as compared to 73% of controls; however, that study did not differentiate between interictal headache and postictal headache.^25^ The prevalence of postictal headache in our current study was similar to what has been reported in the literature which ranges between 4% and 52%.^3^, ^6^, ^8^, ^10^, ^12^, ^14^, ^15^, ^16^, ^17^, ^18^, ^19^, ^20^ Migraine represented the majority of postictal headaches in other series as well as in the present study.

**Table 3:** Review of studies reporting prevalence of interictal and postictal migraine

Authors	Headache present	Interictal migraine	Postictal migraine
Wang, et al,^3^ n = 1109	54.3%	11.7%	NA
Mainieri, et al,^5^ n = 388	53.9%	26.3%	9.5%
Kanemura et al,^6^ n = 98	34% a	NA	NA
Kwan et al,^7^ n = 227	22%	NA	NA
HELP Study Group,^8^ n = 597	NA	12.4%	8.9%
Hofstra, et al,^9^ n = 255	73%	25.5%	NA
Syvertsen, et al,^10^ n = 109	65%	25.6%	18.3%
Schon and Blau,^11^ n = 100	NA	9%	NA
Yankovsky, et al,^12^ n = 100	59%	2%^b^	NA
Gameleira, et al,^13^ n = 304	66.1%^c^	NA	NA
Nunes, et al,^25^ n = 100	92%^d^	NA	NA
Wang, et al,^15^, n = 854	NA	4.9%	23.4%
Botha, et al,^16^ n = 200	54.5%	20%	24.5%
Ito, et al^14^; n = 199	69.3%	NA	NA
Bernasconi, et al,^17^ n = 100	59%	NA	NA
Forderreuther, et al,^18^ n = 126	NA	8.7%	16.7%
Cai, et al,^19^ n = 101	NA	14%	21%
Duchaczek, et al^20^; n = 201	56.2%	10.9%	7%
Total (mean)	58.5% (n = 3400)	13.2% (n = 4140)	16.1% (n = 2576)
Present study	80.8%	40%	31.7%

a Seizure associated headache only.

b Remaining 29% were unclassifiable.

c 32.9% migraine.

d 56% migraine.

The second questionnaire assessing incident postictal headache did not demonstrate any lateralizing value with regard to seizure localization. This finding is limited by the relatively smaller number of postictal headache surveys completed by our cohort. The lower prevalence of migraine in this second questionnaire as compared to the first can likely be attributed to differences in the questionnaire. The second questionnaire could not ask about the duration of the headache due to the fact that we were asking about the headache at the time it was occurring. We could not ask about worsening with physical activity due to most patients being on restrictions during their EMU stay and the fact that they had just had a seizure and would not be doing much physical activity. It could also potentially be due to reporting bias in the retrospectively assessed postictal headaches in the first questionnaire. In the author's review of the literature, however, this is the first published attempt to classify incident postictal headaches at the time of EEG recording. We found two studies supporting an ipsilateral localizing value to postictal headaches in temporal lobe epilepsy^12^, ^17^, and three studies which found no localizing value.^11^, ^18^, ^19^ The studies failing to show localizing values did not separate temporal and extratemporal epilepsies. None of these studies used patient reports at the time of headache during epilepsy monitoring. We performed the second questionnaire in order to decrease the potential for recall bias on part of the patients by assessing the lateralization of postictal headache while patients were actively having a headache.

Headaches, especially migraine, add to the existing morbidity of patients suffering from epilepsy. Headaches and migraine are underdiagnosed and under‐treated in the general population, and our study suggests this is also unfortunately true in PWE.^2^ The costs of both epilepsy and migraine are significant. PWE incur an average of $4593 in excess medical expenses compared to the general population with an estimated annual impact of $9.6 billion dollars.^26^, ^27^ Migraineurs incur over $600 in excess direct medical costs and indirect costs compared over six months to migraine‐free individuals.^28^ Those with severe migraine have even higher costs. Direct costs and disability increase with increasing frequency of headache.^29^ In addition to the financial toll, both conditions have significant psychosocial impact. Poorer quality of life and increased psychiatric diagnoses are seen in both conditions, especially depression and anxiety.^30^, ^31^ Those with epilepsy also have increased risk of panic, suicidal ideation, and bipolar disorder and are more likely to be unemployed or unable to work and live in households with the lowest annual income. Many measures of psychosocial functioning are impaired in migraineurs including emotional and mental health (especially depression and anxiety), work productivity, social functioning, and global measures of disability. There is some evidence that many of these measures improve with appropriate prophylactic treatment of the migraines.^32^

This study continues to support that headaches, predominantly migraine, are very common in epilepsy. It also questions the belief that postictal headache has localizing value for ictal onset. It is the opinion of the authors that epilepsy patients should be asked about headaches, and attempts should be made to adequately treat these headaches to improve quality of life. The generalizability of this study is somewhat limited due to the fact that this was conducted in a tertiary referral center, the self‐reported nature of the questionnaire without clinical interview by a headache expert for validation, and the lack of a control group.

## CONFLICT OF INTERESTS

MW, AM, TB, and JW all report no disclosures. There was no funding for this study. We confirm that we have read the Journal's position on issues involved in ethical publication and affirm that this report is consistent with those guidelines.

## AUTHOR CONTRIBUTIONS

Mark A Whealy, MD, involved in design and conceptualization of the study, analyzed the data, interpreted the data, and drafted the manuscript for intellectual content; Anna Myburgh, APRN, MS, CNP, involved in design and conceptualization of the study and acquisition of data, interpreted the data, and revised the manuscript for intellectual content; Tanya J Bredesen, APRN, MSN, CNP, involved in acquisition of data and revised the manuscript for intellectual content; Jeffrey W Britton, MD, involved in design and conceptualization of the study, interpreted the data, and revised the manuscript for intellectual content.
