# Supplementary figures and images for: Headache in epilepsy: A prospective observational study
Source: Epilepsia Open. 2019 Oct 21;4(4):593–8. doi: 10.1002/epi4.12363 (PMC6885695; doi:10.1002/epi4.12363)

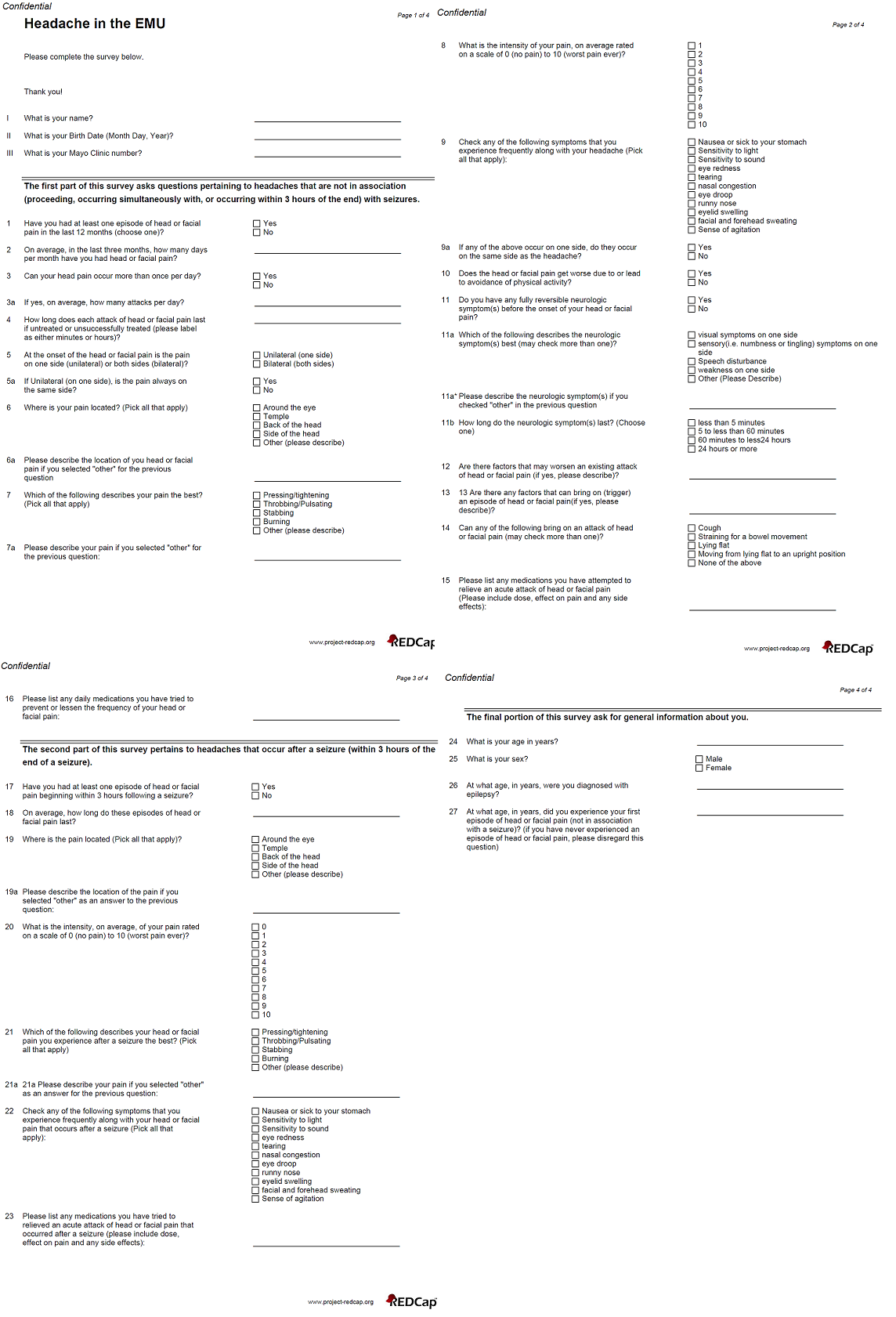

Supplement: Supplementary file 1 [file EPI4-4-0-s001.tif]

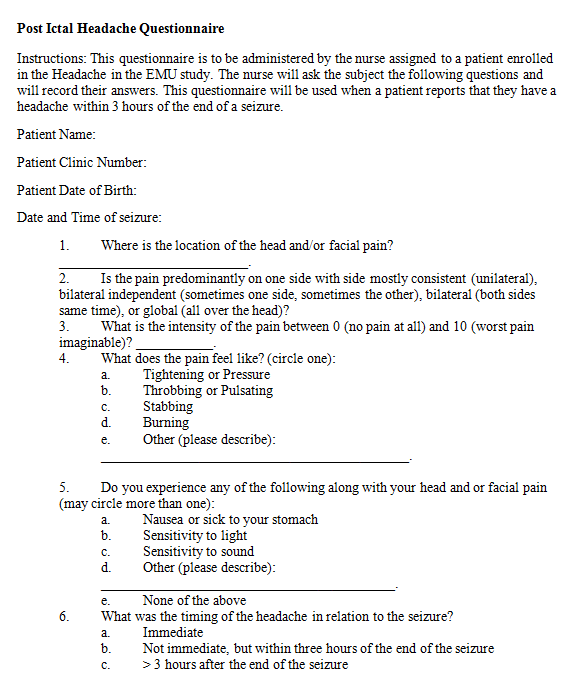

Supplement: Supplementary file 2 [file EPI4-4-0-s002.tif]
